# Supplementary material for: Genome-Wide Identification and Expression Analysis of Expansin Gene Family in the Storage Root Development of Diploid Wild Sweetpotato Ipomoea trifida
Source: Genes (Basel). 2022 Jun 10;13(6):1043. doi: 10.3390/genes13061043 (PMC9222398; doi:10.3390/genes13061043)
Supplement: Supplementary file 1 [file genes-13-01043-s001.zip › genes-1745337-supplementary.pdf]

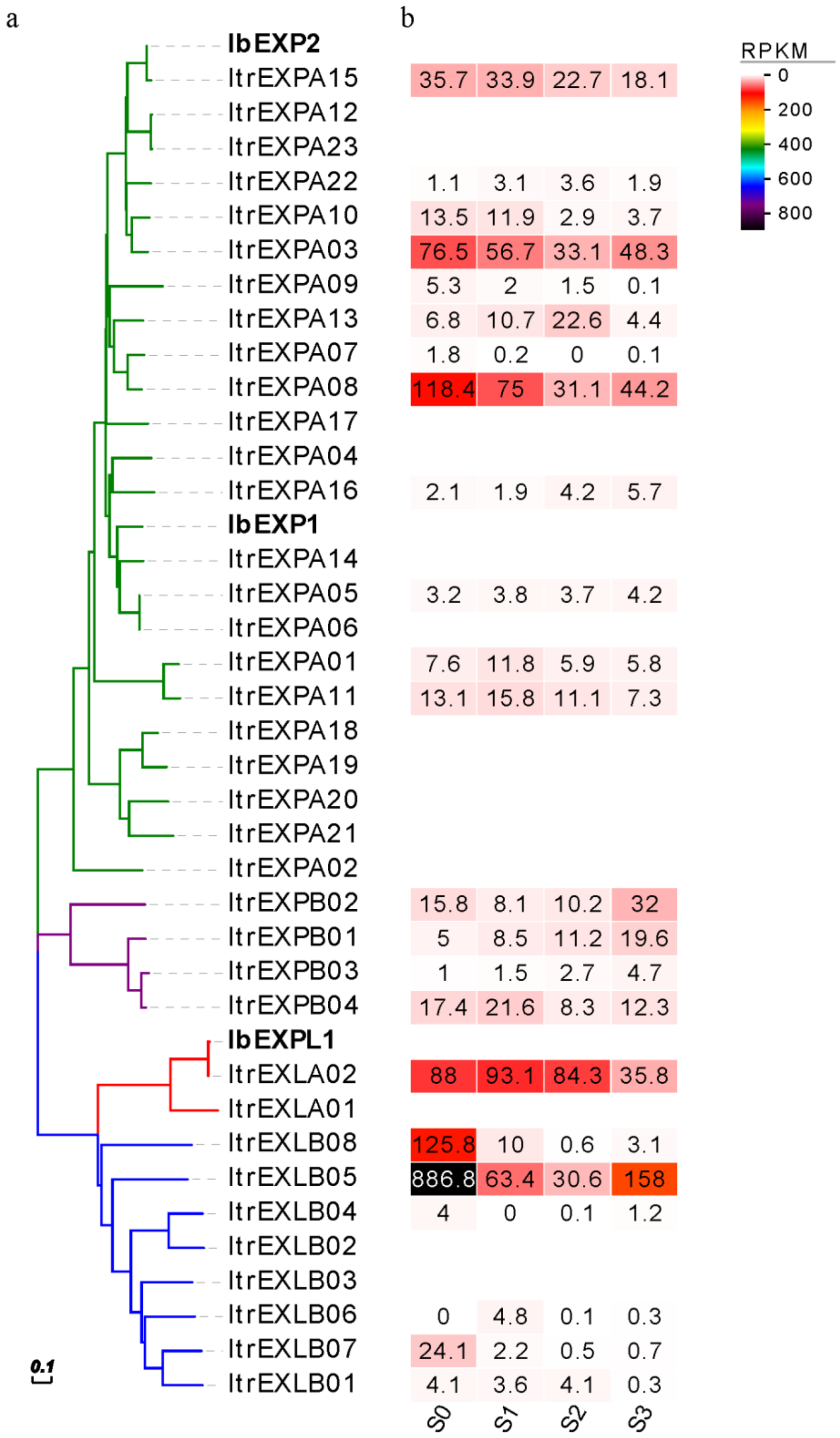

Figure S1. The phylogenetic relationships of expansin proteins between sweetpotato and Y22. (**a**): The phylogenetic relationships between IbEXP1, IbEXP2, IbEXPL1 and Y22 expansin proteins. (**b**): The expression heatmap of the Y22 expansin genes (RPKM > 1).

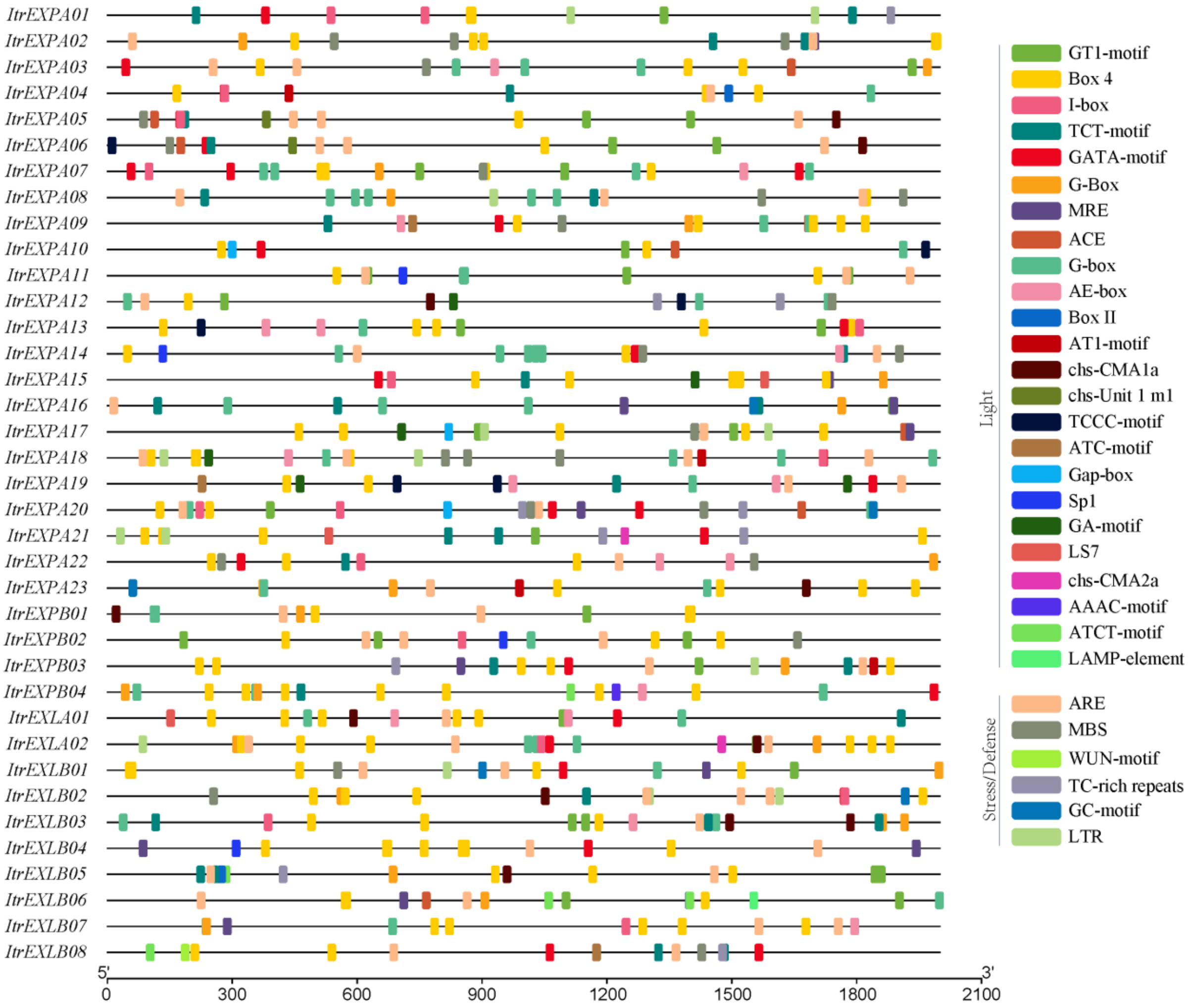

Figure S2. Potential light and stress/defense *cis*-elements in the promoter region of expansin genes. Left part showed the locations of *cis*-elements in the promoter regions, right part showed the *cis*-element types and their symbols.

Table S1. The primer pairs of selected genes for qRT-PCR.

Gene Name	Primer Name	5'-3' sequence	Products
ItrEXPA10	F-PA10	CTCGCCGTCCAAGCCAGAAT	120bp
	R-PA10	CCCATACCCACAAGCACCAC	
ItrEXPA13	F-PA13	GCCCTCCGAACTATGCTTTGC	167bp
	R-PA13	CCTTATGCCTCCTTTCCTCACAC	
ItrEXPA03	F-PA03	TACGCCTTGCCTAATGACAATG	177bp
	R-PA03	CTAGGTTGAAGAAACGGTGGC	
ItrEXPA15	F-PA15	TGTGGAGCCTGCTTTGAGATC	166bp
	R-PA15	GCATGGCGAGATCAAAATGCG	
ItrEXPA09	F-PA09	ATCCAACGCATACCTAACGG	116bp
	R-PA09	CGAAAGTCTGCCCAAATCTCCAA	
ItrEXPA08	F-PA08	CAACACTTCGACATGGCTCAG	164bp
	R-PA08	CCAACGTTAGTCACCAACACC	
ItrEXPB02	F-PB02	AGTGCATTTCGACCTCAGCGG	185bp
	R-PB02	CCACCAAGAGTGAAAGCCAGAA	
ItrEXPB03	F-PB03	ATGCTCGGGAAAGGCAGTGAC	127bp
	R-PB03	CTGGTCAGCTTTGCCGGAAGA	
ItrEXLA02	F-LA02	CTGCGTCCACCAAACCAAAGTC	116bp
	R-LA02	CTGCGGCAGCAAGATGTCCT	
ItrEXLB05	F-LB05	GGAGCTTGTGGGTTTGGTGAC	151bp
	R-LB05	TCCTCGTACCTTCCTCGTTGC	
ItrEXLB08	F-LB08	GTCTTGCAGTTCTCATAGGGAC	127bp
	R-LB08	CTATCTGTGCCATTGTCATCGG	

Table S2. The expansin genes of *I. nil* and *I. triloba*.

I. nil		*I. triloba*	
**Gene Name**	**Gene ID**	**Gene Name**	**Gene ID**
IniEXPA01	gene-LOC109162042	*IlbEXPA01*	gene-LOC115998052
IniEXPA02	gene-LOC109166240	*IlbEXPA02*	gene-LOC116025128
IniEXPA03	gene-LOC109189038	*IlbEXPA03*	gene-LOC116020824
IniEXPA04	gene-LOC109193479	*IlbEXPA04*	gene-LOC115999375
IniEXPA05	gene-LOC109154062	*IlbEXPA05*	gene-LOC116000481
IniEXPA06	gene-LOC109154072	*IlbEXPA06*	gene-LOC115999911
IniEXPA07	gene-LOC109154073	*IlbEXPA07*	gene-LOC116007517
IniEXPA08	gene-LOC109181060	*IlbEXPA08*	gene-LOC116003819
IniEXPA09	gene-LOC109173867	*IlbEXPA09*	gene-LOC116013951
IniEXPA10	gene-LOC109186551	*IlbEXPA10*	gene-LOC116020110
IniEXPA11	gene-LOC109154973	*IlbEXPA11*	gene-LOC116019994
IniEXPA12	gene-LOC109154972	*IlbEXPA12*	gene-LOC115998007
IniEXPA13	gene-LOC109157987	*IlbEXPA13*	gene-LOC116030898
IniEXPA14	gene-LOC109159018	*IlbEXPA14*	gene-LOC116017466
IniEXPA15	gene-LOC109148147	*IlbEXPA15*	gene-LOC116003780
IniEXPA16	gene-LOC109186601	*IlbEXPA16*	gene-LOC115999171
IniEXPA17	gene-LOC109192973	*IlbEXPA17*	gene-LOC116011772
IniEXPA18	gene-LOC109173472	*IlbEXPA18*	gene-LOC116022872
IniEXPA19	gene-LOC109149167	*IlbEXPA19*	gene-LOC116027442
IniEXPA20	gene-LOC109152686	*IlbEXPA20*	gene-LOC116026901
IniEXPA21	gene-LOC109183517	*IlbEXPA21*	gene-LOC116001114
IniEXPA22	gene-LOC109168111	*IlbEXPA22*	gene-LOC116012277
IniEXPA23	gene-LOC109188880	*IlbEXPA23*	gene-LOC115998240
IniEXPA24	gene-LOC109167477	*IlbEXPA24*	gene-LOC116003784
IniEXPA25	gene-LOC109177514	*IlbEXPA25*	gene-LOC116030964
IniEXPA26	gene-LOC109188155	*IlbEXPA26*	gene-LOC116028302
IniEXPA27	gene-LOC109153624	*IlbEXPA27*	gene-LOC116030858
IniEXPA28	gene-LOC109153727	*IlbEXPB01*	gene-LOC116008214
IniEXPA29	gene-LOC109191378	*IlbEXPB02*	gene-LOC116017748
IniEXPA30	gene-LOC109153593	*IlbEXPB03*	gene-LOC116014045
IniEXPA31	gene-LOC109153631	*IlbEXPB04*	gene-LOC116014258
IniEXPB01	gene-LOC109188048	*IlbEXPB05*	gene-LOC116014428
IniEXPB02	gene-LOC109186602	*IlbEXPB06*	gene-LOC116014343
IniEXPB03	gene-LOC109186593	*IlbEXLA01*	gene-LOC116019640
IniEXPB04	gene-LOC109193325	*IlbEXLA02*	gene-LOC116029340
IniEXPB05	gene-LOC109193328	*IlbEXLB01*	gene-LOC116028927
IniEXPB06	gene-LOC109193326	*IlbEXLB02*	gene-LOC116002815
IniEXPB07	gene-LOC109193320	*IlbEXLB03*	gene-LOC115997228
IniEXLA01	gene-LOC109161325	*IlbEXLB04*	gene-LOC116001079
IniEXLB01	gene-LOC109174806	*IlbEXLB05*	gene-LOC116001309
IniEXLB02	gene-LOC109177426	*IlbEXLB06*	gene-LOC116028391

Table S2. The expansin genes of *I. nil* and *I. triloba* (continued).

I. nil		*I. triloba*	
**Gene Name**	**Gene ID**	**Gene Name**	**Gene ID**
IniEXLB03	gene-LOC109174872	*IlbEXLB07*	gene-LOC116001078
IniEXLB04	gene-LOC109185131	*IlbEXLB08*	gene-LOC116002898
IniEXLB05	gene-LOC109185314	*IlbEXLB09*	gene-LOC116030832
IniEXLB06	gene-LOC109174855	*IlbEXLB10*	gene-LOC116030871
IniEXLB07	gene-LOC109174854		

The expansin genes were identified by the same pipeline as that used for Y22.

Table S3. The duplication type of expansin genes in Y22 genome.

Subfamily	Gene Name	Gene Id	Code	Type of duplication
EXPA	*ItrEXPA01*	Itr.Sc0000011.164	4	WGD or segmental
EXPA	*ItrEXPA02*	Itr.xfSc0000576.12	4	WGD or segmental
EXPA	*ItrEXPA03*	Itr.Sc0000007.290	4	WGD or segmental
EXPA	*ItrEXPA04*	Itr.Sc0000051.50	4	WGD or segmental
EXPA	*ItrEXPA05*	Itr.Sc0000046.34	4	WGD or segmental
EXPA	*ItrEXPA06*	Itr.Sc0000046.36	3	Tandem
EXPA	*ItrEXPA07*	Itr.xfSc0000007.14	4	WGD or segmental
EXPA	*ItrEXPA08*	Itr.xfSc0000007.15	3	Tandem
EXPA	*ItrEXPA09*	Itr.Sc0000078.10	4	WGD or segmental
EXPA	*ItrEXPA10*	Itr.Sc0000001.78	2	Proximal
EXPA	*ItrEXPA11*	Itr.xfSc0000049.46	4	WGD or segmental
EXPA	*ItrEXPA12*	Itr.Sc0000003.235	4	WGD or segmental
EXPA	*ItrEXPA13*	Itr.Sc0000003.134	4	WGD or segmental
EXPA	*ItrEXPA14*	Itr.Sc0000025.53	4	WGD or segmental
EXPA	*ItrEXPA15*	Itr.Sc0000014.125	4	WGD or segmental
EXPA	*ItrEXPA16*	Itr.Sc0000087.3	4	WGD or segmental
EXPA	*ItrEXPA17*	Itr.Sc0000013.190	4	WGD or segmental
EXPA	*ItrEXPA18*	Itr.xpSc0079065.36	4	WGD or segmental
EXPA	*ItrEXPA19*	Itr.xpSc0079065.38	3	Tandem
EXPA	*ItrEXPA20*	Itr.xpSc0079065.39	3	Tandem
EXPA	*ItrEXPA21*	Itr.xpSc0079065.40	3	Tandem
EXPA	*ItrEXPA22*	Itr.xfSc0000000.33	4	WGD or segmental
EXPA	*ItrEXPA23*	Itr.xfSc0001203.1	1	Dispersed
EXPB	*ItrEXPB01*	Itr.xfSc0000185.16	4	WGD or segmental
EXPB	*ItrEXPB02*	Itr.Sc0000024.30	4	WGD or segmental
EXPB	*ItrEXPB03*	Itr.xfSc0000047.33	4	WGD or segmental
EXPB	*ItrEXPB04*	Itr.xfSc0001448.1	1	Dispersed
EXLA	*ItrEXLA01*	Itr.Sc0000050.70	4	WGD or segmental
EXLA	*ItrEXLA02*	Itr.Sc0000020.14	4	WGD or segmental
EXLB	*ItrEXLB01*	Itr.xpSc0079072.22	4	WGD or segmental
EXLB	*ItrEXLB02*	Itr.xfSc0000061.14	4	WGD or segmental
EXLB	*ItrEXLB03*	Itr.xfSc0000061.13	3	Tandem
EXLB	*ItrEXLB04*	Itr.xfSc0000239.16	1	Dispersed
EXLB	*ItrEXLB05*	Itr.Sc0000071.6	4	WGD or segmental
EXLB	*ItrEXLB06*	Itr.xfSc0000002.83.1	4	WGD or segmental
EXLB	*ItrEXLB07*	Itr.xfSc0000002.86	3	Tandem
EXLB	*ItrEXLB08*	Itr.xfSc0000002.103	4	WGD or segmental

Comparative genomic analysis revealed that the WGT occurred in an ancient ancestor of *Ipomoea* [23, 24], therefore the "WGD" in this table was the WGD or WGT.

Table S4. Collinearity result of expansin genes between *C. canephora* and Y22 genome.

Location	*C. canephora* Genes	*I. trifida* Genes				
		iter=1	iter=2	iter=3	iter=4	iter=5
chr1	*gene-GSCOC_T00028253001*	*ItrEXPA15*	*ItrEXPA12*		*ItrEXPA10*	
chr1	*gene-GSCOC_T00016583001*		*ItrEXPA13*	*ItrEXPA17*		
chr2	*gene-GSCOC_T00028856001*					
chr2	*gene-GSCOC_T00028854001*					
chr2	*gene-GSCOC_T00029906001*	*ItrEXPA03*	*ItrEXPA10*		*ItrEXPA12*	
chr4	*gene-GSCOC_T00035723001*	*ItrEXPA04*				
chr5	*gene-GSCOC_T00042204001*	*ItrEXPB02*				
chr6	*gene-GSCOC_T00023514001*		*ItrEXPA22*		*ItrEXPA15*	*ItrEXPA12*
chr6	*gene-GSCOC_T00042971001*		*ItrEXPA18*			
chr7	*gene-GSCOC_T00039583001*					
chr7	*gene-GSCOC_T00036829001*		*ItrEXPA11*			
chr8	*gene-GSCOC_T00027140001*			*ItrEXLB03*		
chr8	*gene-GSCOC_T00027139001*			*ItrEXLB03*		
chr8	*gene-GSCOC_T00027142001*	*ItrEXLB06*		*ItrEXLB03*		
chr8	*gene-GSCOC_T00027143001*	*ItrEXLB07*		*ItrEXLB03*		
chr8	*gene-GSCOC_T00000261001*	*ItrEXLB08*				
chr8	*gene-GSCOC_T00037639001*	*ItrEXLB08*				
chr8	*gene-GSCOC_T00030309001*					
chr8	*gene-GSCOC_T00027141001*			*ItrEXLB02*		
chr9	*gene-GSCOC_T00041034001*	*ItrEXLA02*				
chr10	*gene-GSCOC_T00024595001*		*ItrEXPA09*			
scaffold_368	*gene-GSCOC_T00003973001*					

All of the identified expansin genes of *C. canephora* are shown in this table; the term 'iter' was the software parameter.

Table S5. The protein sequences of different conserved motifs.

Motif	Length	Motif Consensus	PFAM Domain
1	21	ALSTALFNNGLSCGACYEVKC	DPBB_1
2	15	WIPMSRNWGQNWETN	Pollen_allerg_1
3	15	AGIVPVEYRRVPCKK	DPBB_1
4	23	AHATFYGGSDASGTMGGACGYGN	DPBB_1
5	27	DNGGWCNPPRPHFDLSMPAFLKIAQYR	DPBB_1
6	29	KGGIRFTINGHRYFNLVLVTNVGGAGDVH	
7	29	LSFRVTTSDGRTVTSYNVAPANWQFGQTF	
8	21	GPGSITVTATNFCPPNYALPN	DPBB_1
9	11	LYSQGYGTNTA	
10	15	TDFILSYTAFGKMAK	DPBB_1
11	21	KENALCSEKGVKVVITDEGEG	DPBB_1
12	21	KILDSSSNPNYLAIVILNQGG	Pollen_allerg_1
13	21	SSKNAIPADWKAGVTIDTGIQ	
14	8	VSIKGSRT	
15	34	WKEAHATYYGPPEGTIGGACGFEEYKQTYSPYTA	DPBB_1
